# Is the six-minute walk test still reliable compared to cardiopulmonary exercise test for exercise capacity in children with congenital heart disease?

**DOI:** 10.3389/fped.2022.965739

**Published:** 2022-11-14

**Authors:** Jiangbo Qu, Hui Shi, Yugong Guo, Xinxin Chen, Xuwen Xiao, Xiaojuan Zheng, Yanqin Cui

**Affiliations:** ^1^ Cardiac Intensive Care Unit, Heart Center, Guangzhou Women and Children's Medical Center, Guangzhou Medical University, Guangzhou, China; ^2^ Department of Biostatistics and Epidemiology, School of Public Health, Sun Yat-sen University, Guangzhou, China

**Keywords:** congenital heart diasease, six minute walk test, cardiopulmonary exercise test (CPET), children, six minute walk distance

## Abstract

**Objectives:**

We aimed to assess the validity of the six-minute walk test (6MWT) to reflect the functional capacity of children with congenital heart disease (CHD), evaluate a possible correlation between the 6MWT distance with cardiopulmonary exercise test (CPET) variables, as well as to find a cutoff value to stratification the physical fitness in this population.

**Methods:**

We enrolled 459 children with CHD, 6–18 years old, who performed a complete CPET and 6MWT on the same day in a cross-sectional observational study. Correlations between variables of CPET and six-minute walking distance (6MWD) were analyzed and cutoff values of 6MWD were identified for the classification of the physical fitness in the population.

**Results:**

The mean distance ambulated during the 6MWT was 578 ± 65 m, 590 ± 65 m for boys, and 562 ± 62 m for girls (*p* < 0.001). Both VO_2max_ and %predicted VO_2max_ showed a correlation with the 6MWT distance (*r* = 0.35, *p* < 0.001 and *r* = 0.51, *p* < 0.001, respectively), and an inverse correlation was found between VE/VCO2 slope and the 6MWT distance (*r* = −0.31; *p* < 0.001). There appeared to be a linear association between 6MWD and VO_2max_ up to a 6MWD of approximately 600 m. We divided the population into 4 subgroups (boys <130 cm; boys ≥130 cm; girls <130 cm; girls ≥130 cm), and get the cutoff values (554 m, 617 m, 549 m, 587 m) respectively equivalent to 80% of predicted VO_2max_. The 6MWT distances of another 102 patients were applied for external verification of the cutoff values.

**Conclusions:**

Our study provided evidence on when a 6MWT should be considered as a convincing complementary test in the pediatric population with CHD and explored the classification of exercise tolerance using a 6MWD value. The cut-off values for 6MWD may be qualified as an intervention target for exercise rehabilitation.

## Introduction

Although a significant increase in survival of children with congenital heart disease (CHD) has been observed with advances in cardiac surgical techniques and perioperative support in decades, decision-making of interventions or identifying deteriorating conditions in long-term follow-up are still critical issues for complex CHD patients (1, 2). Many studies recommended that physical activity should be assessed routinely as part of clinical follow-up in adult and young patients with CHD (3–5).

Objective assessment of the cardiopulmonary fitness is one of the most important factors affecting the quality of life of patients and prognosis after surgical correction (3, 6, 7), which remains difficult in pediatric clinical practice. The gold standard for cardiopulmonary fitness expression is the maximum oxygen uptake (VO_2max_) obtained at peak exercise of cardiopulmonary exercise testing (CPET), which is an established and reliable indicator of physical fitness (8), but expensive, requiring sophisticated equipment and specialized personnel, cannot be widely conducted in the primary or community health care facilities, especially in developing countries. Moreover, CPET does not represent the usual physical activity level of these children. Therefore, CPET has not yet been used widely in the follow-up of patients with CHD, especially in children and adolescents.

As a simple, reproducible, negligible cost and safe exercise test (9), the six-minute walk test(6MWT) measures the distance a participant can walk within 6 min. It was more likely to be easily used to quantify the functional capacity of patients, which closely reflects the activities in daily life because of the submaximal nature of the test (9–11).

Previous studies in patients with pulmonary arterial hypertension (PAH), chronic heart failure and grown-up patients with CHD (12–16) provided the correlation between walking distance in 6 min and VO_2max_. Despite widespread use, the role of the 6MWT in the evaluation of children with CHD remains lacking so far, and more data and research regarding the application in this population are required. In this article, we tried to assess the validity of 6MWT to reflect the functional capacity of children and adolescents with CHD, evaluate a possible correlation between the distance walked during the 6MWT with CPET variables in measuring exercise capacity, as well as to find a cutoff value to stratification the physical fitness in this population.

## Methods

### Study subjects

This cross-sectional observational study was carried out from October 2018 to March 2020 in our CPET laboratory, and a total of 459 children with congenital heart defects aged 6–18 years were included in this study and performed CPET assessment and 6MWT, as a part of routine pediatric cardiology outpatient follow-up. In addition to the majority of the subjects having previously undergone corrective surgical interventions, fewer preoperative patients were also included. Our study population was provided in Table 1. Of these, 76 patients underwent the surgery with Fontan physiology and 16 patients had no surgery. No CHD patients were in New York Heart Association (NYHA) class IV.

**Table 1:** Primary diagnostic categories of the study population.

Classification of congenital heart disease	*N*
Left to right shunts corrected	
Septal defects	123 (26.8%)
PDA	9 (2.0%)
Fontan circulation	
Single ventricle	34 (7.4%)
ccTGA	26 (5.7%)
Pulmonary atresia	7 (1.5%)
Double outlet of right ventricle	4 (0.9%)
Ebstein anomaly	2 (0.4%)
Complex anatomy, biventricular corrected	
TOF	37 (8.1%)
TGA	26 (5.7%)
ccTGA	5 (1.1%)
Double outlet of right ventricle	16 (3.5%)
Coarctation of the aorta	5 (1.1%)
Total anomalous pulmonary venous connection	16 (3.5%)
Truncus arteriosus communis	4 (0.9%)
Anomalous coronary artery from the pulmonary artery	12 (2.6%)
Pulmonary atresia and extremely severe pulmonary stenosis	59 (12.9%)
Ebstein anomaly	2 (0.4%)
Severe valvular malformation	27 (5.9%)
Others^a^	45 (9.8%)
All cases	459

CHD, congenital heart disease; VSD, ventricular septal defect; ASD, atrial septal defect; TGA, d-transposition of the great artery; ccTGA, congenitally corrected transposition; TOF, Tetralogy of Fallot; PDA, Patent ductus arteriosus.

^a^ Coronary anomalies, mild or moderate valvular defects, atrio-ventricular canal, left to right uncorrected, several congenital heart defects in the same patient.

Participants were not included in the study if they had contraindications (fever, respiratory failure, uncontrolled asthma, acute myocarditis or pericarditis, uncontrolled severe arrhythmias, uncontrolled heart failure, and children suffering from genetic defects leading to inability to cooperate or noncompliance, or had any other chronic medical condition other than their known heart disease, or with obesity.

The study protocol was approved by the Medical Ethics Committee of Guangzhou Women and Children's Medical Center, and informed consent was provided by all patients before exercise testing.

### Procedures

The clinical data collected included gender, date of birth, body mass (BM) (kg) and body height (cm), surgery or not, age at surgery, medical treatments, and type of cardiac surgery. Functional capacity was graded according to the NYHA class.

Plasma NT-proBNP for CHD patients was measured within 3 h before beginning the cardiopulmonary exercise test and the 6-min walk test, using a commercially available fluorescence immunoassay (competitive Enzyme Immuno Assay; ReLIA II, Shenzhen, China) by the Central Laboratory Institute, Guangzhou Women and Children's Medical Center.

All children underwent CPET and 6MWT without complications on the same day, and the interval between tests was more than 60 min.

### Cardiopulmonary exercise testing

All participants underwent a symptom limited CPET after performing spirometry first, using a treadmill (GE Healthcare, Little Chalfont, UK) with a breath-by-breath respiratory gas exchange analysis (MasterScreen CPX, Jaeger, Vyaire, Germany) according to the ramped Bruce protocol, as suggested by the American College of Sports Medicine (ACSM). The test was terminated when the participants demonstrated subjective unbearable symptoms, or when they attained maximal exertion despite intense verbal encouragement. An incremental overall duration between 6 and 12 min was obtained with this protocol.

Blood pressure (BP), pulse oximetry and heart rate (HR) were monitored and recorded for the duration of the test. Oxygen uptake (VO_2_), carbon dioxide consumption (VCO_2_) and minute ventilation (VE) were measured through the respiratory gas exchange analysis. The ventilatory threshold (VAT) was derived during maximal exercise testing, and determined by use of the modified V-slope method and reinforced by the VE/VO_2_ curve.

The following criteria for reaching VO_2max_ were used: (1) respiratory exchange ratio (RER) > 1.1(2) peak HR > 85% of age-predicted maximum; (3) plateau of VO_2_ can be seen despite increasing the exercise intensity, or the peak VO_2_ deﬁned as the highest mean VO_2_ of any 30 s interval during exercise was informed without a VO_2_ plateau (17). VO_2max_ and VAT values were normalized in a percentage of the predicted VO_2max_ according to normal values from Wasserman and Cooper (18).

### The 6-minute walking test

The 6MWT was performed following a standard protocol proposed by the American Thoracic Society guidelines (19). The subjects were instructed to walk back and forth along a flat, 30-m long corridor as much as possible for 6 min. The total distance walked in 6 min was measured, with interruption or slowing down the rhythm if necessary. All children and adolescents received the same instructions before undertaking the walk test. The heart rate, pulse oximetry, blood pressure and respiratory rate were measured at the beginning and end of the test, as the modified BORG scale, used to assess the subjective sensation of dyspnea and fatigue of the lower limbs. The walked distance during the test was compared with reference values with the equation proposed by Li (20).

### Data statistics

The study population was described with means and standard deviations (SD) for quantitative variables and with frequencies for qualitative variables. Categorical data were analyzed using the chi-square or Fisher's exact test, however continuous data used the independent samples *t*-test or Wilcoxon rank-sum test where appropriate.

Locally weighted regression (LOWESS smoothing) was used to further determine the relationship between 6MWT distance and VO_2max_.

Additionally, we performed Receiver Operating Characteristic (ROC) analysis to determine a cutoff value for the 6MWT that corresponds to 80% of predicted VO_2 max_, as VO_2max_ ≥80% of predicted value were regarded to have a preserved exercise capacity (21, 22). Since our study showed that 6MWT was associated with gender and height, we divided the population into four subgroups (boys with a height of <130 cm; boys with a height of ≥130 cm; girls with a height of <130 cm; girls with a height ≥130 cm) and performed separate ROC analyses for the different subgroups. We then derived cutoff values for the four different subgroups to improve practical applicability, and finally validated the results using external validation.

## Results

### Patient's baseline characteristics

Baseline characteristics of all patients (*n* = 459) are shown in Table 2. In total, 56.2% were male and 43.8% were female, and the median age was 8.76 years old (range 7–11 years). In our subjects 76.3% of the patients were NYHA class I and only 0.3% were NYHA class III.

**Table 2:** Patient's baseline characteristics.

	All case (*n* = 459)
Age (year)	8.8 (7.0-10.8)
Gender (%)	
Male	258 (56.2%)
Female	201 (43.8%)
Height (cm)	127.0 (118.0-141.5)
Weight (kg)	24.5 (20.3-31.7)
BMI (kg/m^2^)	15.2 (14.0-16.6)
BSA (m^2^)	0.9 (0.8-1.1)
NYHA (%)	
I	350 (76.3%)
II	106 (23.1%)
III	3 (0.7%)
NT-proBNP	246.7 (130.4–677.7)
Therapy	
Surgery	431 (93.9%)
Without surgery	28 (6.1%)

BMI, Body Mass Index; BSA, body surface area; NYHA, New York Heart Association; Values are presented as interquartile range (IQR).

### Correlations between variables of CEPT and 6MWT

The cardiopulmonary responses during CPET and 6MWT distance are summarized in Table 3. The mean VO_2max_ and VO_2max_ (%pred) were 36.23 ± 6.87 ml/min/kg and 0.81 ± 0.17 respectively. There were 270 (58.8%) patients who had VO_2max_ more than 80% of predictive value. No complications occurred during maximal exercise testing and all tests were terminated because of dyspnoea (38%) or fatigue (62%). The mean distance ambulated during the 6MWT was 578 ± 65 m (range from 390 to 762 m), with no patient requiring a rest stop, which represents approximately 83% of the mean value of predicted distance (695 ± 59 m) by the formula.

**Table 3:** Exercise performance and the relationship between peak oxygen uptake and the 6-min walk test.

Variables	Value	6MWT
Cardiopulmonary exercise testing		
VO_2max_ (%pred)	0.81 ± 0.17	*r* = 0.35^b^
VO_2max_ (ml/min/kg)	36.2 ± 6.9	*r* = 0.51^b^
VO_2_/kg @ AT (ml/min/kg)	25.1 ± 4.8	*r* = 0.14^a^
O_2_/HR (ml/beat)	5.5 ± 1.9	*r* = 0.57^b^
VE/VCO_2_ slope	34.7 ± 8.0	*r* = −0.31^b^
RER	1.14 ± 0.98	*r* = 0.16^b^
OUES	1051.52 ± 358.06	*r* = 0.56^b^
6MWT distance (m)	578 ± 65	—

Data are presented as means ± SD.

^a^ <0.05.

^b^ <0.001.

VO_2max_, peak oxygen uptake; AT, anaerobic threshold; VE/VCO_2_, ventilatory equivalent of carbon dioxide; HR, heart rate; O2/HR, O2 pulse; OUES, oxygen uptake efficiency slope; 6MWT, The 6-minute walking test; RER, respiratory exchange ratio.

**Figure 1:**
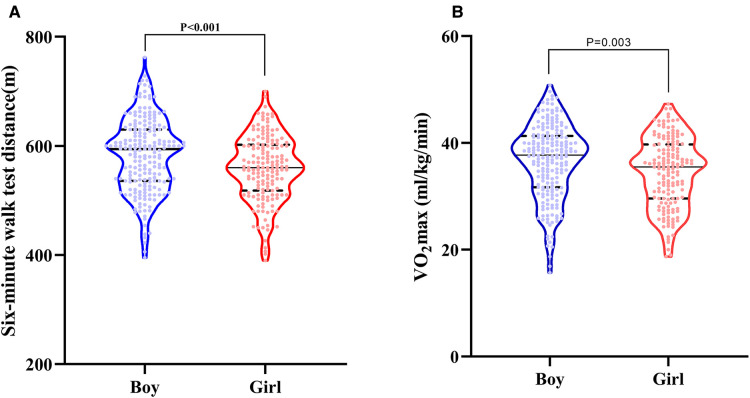
Violin diagrams of the distribution of six-minute walk test (6MWT) distance by gender (**A**); violin diagrams of the distribution of VO_2_ max by gender (**B**). The upper and lower dashed lines represent the first and third quartiles, and the solid line in the middle represents the median.

**Figure 2:**
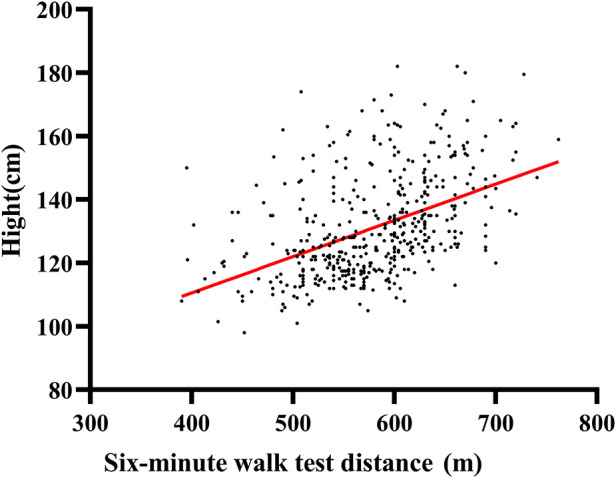
Scatter plots of the relationship between the six-minute walk test (6MWT) distance and height of children and adolescents with congenital heart disease. The red straight line in the scatter plots is linear regression fits.

Univariate correlation between 6MWD and the various demographic in Supplementary Tables S1. The mean 6MWT distance of boys was 590 ± 65 m, higher than girls (562 ± 62 m, *p* < 0.001). Height has the best correlation with 6MWT distance on univariate analysis (all cases, *r* = 0.460, *p* < 0.001; boys, *r* = 0.424, *p* < 0.001; girls, *r* = 0.499, *p* < 0.001). The distribution of 6MWD by gender is shown in Figure 1. The association between height and 6MWD is shown in Figure 2.

Both VO_2max_ and VO_2max_ (%pred) showed a correlation with the 6MWT distance (*r* = 0.35; *p* < 0.001 and *r* = 0.51, *p* < 0.001, respectively), and an inverse correlation was found between VE/VCO_2_ slope and the 6MWT distance (*r* = −0.31; *p* < 0.001).

**Figure 3:**
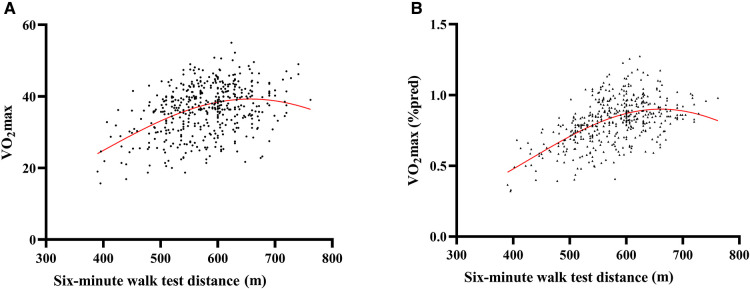
Scatter plots of the relationship between the six-minute walk test (6MWT) distance and VO2max of children and adolescents with congenital heart disease(**A**); scatter plots of the relationship between the six-minute walk test (6MWT) distance and VO_2__max_ (%pred) of children and adolescents with congenital heart disease (**B**). The red curvy lines in the scatter plots are nonparametric regression fits.

However, when the relationship between the 6MWT distance and VO_2max_ was investigated further using locally weighted polynomial regression (lowess), it became apparent that a linear correlation between 6MWT distance and VO_2max_ existed only at low levels of exercise capacity. As illustrated in Figure 3A, there appeared to be a close to a linear association between 6MWT distance and VO_2max_ up to a 6MWT distance of approximately 600 m. A similar phenomenon is also reflected in the relationship between 6MWT distance and VO_2max_ (%pred), see Figure 3B.

We further analyzed the correlation between CPET variables and 6MWT distance in participants with VO_2max_ (%pred) ≥ 80% and <80% separately (Supplementary Table S3). VO_2max_ was 39.7 ± 5.2 ml/min/kg in the group with higher VO_2max_ (%pred) as compared to a VO_2max_ of 31.2 ± 5.8 ml/min/kg in the other group. In children with VO_2max_ (%pred) < 80%, the 6MWT distance correlated more strongly with VO_2max_ [VO_2max_ (%pred): *r* = 0.17, *p* = 0.035; VO_2max_ (ml/min/kg): *r* = 0.34, *p* = 0.001], whereas for the subgroup with VO_2max_ (%pred) ≥ 80%, the correlation between VO_2max_ and 6MWT seemed to be negligible. Similarly, we fitted a plot of the relationship between VO_2max_ and 6MWT distance for both groups using lowess, and the results were consistent with those without grouping, both indicating 600 m as the turning point (Supplementary Figure S1).

### Cutoff values of 6MWT distance for the exercise tolerance

**Figure 4:**
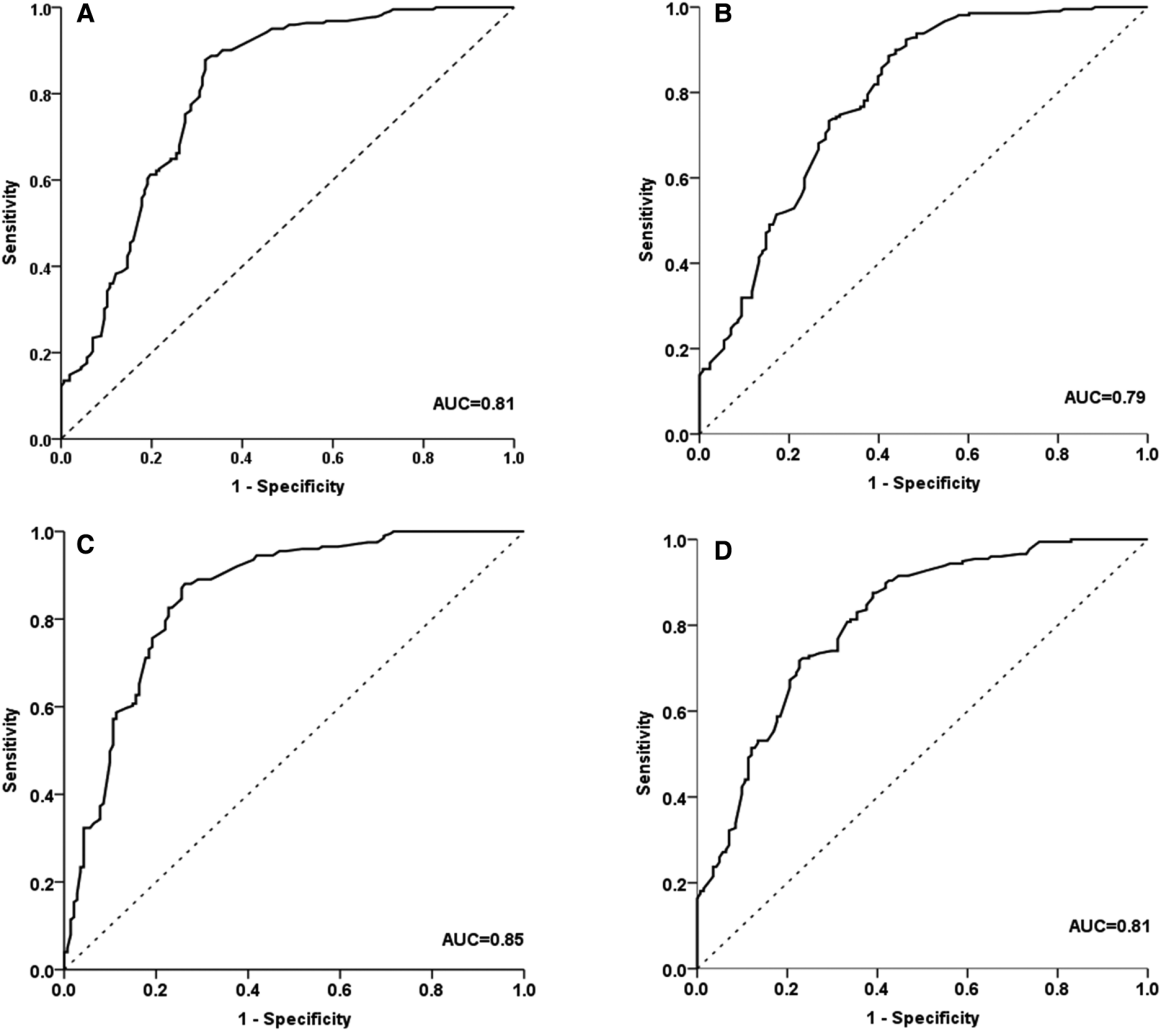
ROC curve analysis for determining six-minute walk test (6MWT) distance cut-off values in boys with a height of <130 cm (**A**); in boys with a height of ≥130 cm (**B**); in girls with a height of <130 cm (**C**); girls with a height ≥130 cm (**D**). ROC, receiver operating characteristic.

To evaluate the diagnostic potential of 6MWT for the cardiopulmonary exercise capacity, we performed ROC analysis according to VO_2max_(%pred) ≥80%, as mentioned in the methodology. We analyzed and calculated Area Under Curve (AUC) for four subgroups, and these four subgroups have AUCs of 0.86, 0.80, 0.87, and 0.87, respectively (Figure 4). The cutoff values regarding sensitivity and specificity were calculated with Youden's index, and the cutoff values of 6MWT for four subgroups were 554 m, 617 m, 549 m, 587 m respectively (Table 3).

### External validation

The external validation was performed using data from this hospital that is representative of the external validation set. The confusion matrix applying the cutoff values of the 102 test sets was shown in Supplementary Table S2. Our final showed an overall accuracy of 81.2%, sensitivity (recall) of 75.5%, and specificity of 76.8%. The positive predictive value (PPV), which indicates “precision,” was 78.2%, and the negative predictive value (NPV) was 72.4%. The above results indicated acceptable performance.

## Discussion

The main objectives of this study were to determine the reliability of the 6MWT in assessing the functional capacity of children with CHD and to investigate any potential relationships between the 6MWT distance and CPET characteristics.

In this study, we investigated the 6MWT and CPET-derived variables in the prognostic assessment of children and adolescents with CHD. Our result showed the patients with CHD had a mean VO_2max_ of 81% of predicted value, which is consistent with the fact that most patients self-perceived their exercise endurance was not significantly inferior to their peers since VO_2max_ above 80% of the predicted value was considered approximate or equal to a normal quality of life. Whereas, from a purely data analysis point of view, children with CHD can still be considered to have depressed VO_2max_ or exercise capacity to some extent compared with healthy children. Our previous study (23) also demonstrated that even for children undergoing simple CHD surgery, their mean value of VO_2max_ was only 91% of the predicted value, compared with 101% for the healthy controls.

Compared with the healthy children of the same age, the 6MWT distance as a submaximal exercise test showed a similar downward trend as VO_2 max_ for children with CHD, given that both accounted for approximately 82% and 81% of the predicted values, respectively. Nevertheless, the criteria for the descending level of the 6MWT distance to evaluate the degree of exercise capacity was controversial and unclear. Dourado (24) and Sperandio (25) et al. verified that a 6MWT distance below 96% is a critical point for identifying physically inactive adults with cardiorespiratory fitness levels below the normal range. Although there are differences in the subjects and statistical methods, it also indicates that the aerobic capacity of children with CHD is distinct from that of the general adult population.

We can expect that the results will be convincing in clinical practice for screening and monitoring the cardiorespiratory risk in children and adolescents with CHD if the 6MWT distance is reported as a percentage of the predicted value. The analyses of 6MWT distance cutoff values have been established to predict outcomes according to the literature (11, 13, 24, 26) on adults with cardiovascular disorders, including chronic heart failure and pulmonary hypertension, and others. In an earlier study by Kehmeier et al. (14) involving 102 grown-up patients with congenital heart disease, a cutoff value of 482 m by 6MWT was shown to be the optimal for identifying a VO_2max_ of ≤15.5 ml/kg/min. However, it is not reasonable for children to utilize a single value of walking distance for the threshold of inadequate cardiopulmonary fitness since 6MWT distance was more likely to be influenced by height and gender for children. We performed the analysis of 6MWT in the children with CHD in comparison with VO_2max_, defined a cutoff value equivalent to 80% of predicted VO_2max,_ and performed external validation further using additional data. To the best of our knowledge, this is the first study to obtain the cutoff value of 6MWT distance in children with CHD by sex and body height. Meanwhile, we also identified that 130 cm as an inflection point of the correlation between height and other variables, had excellent sensitivity and specificity for metrics of grouping.

We observed that the correlation between the 6MWT distance and VO_2max_ was not linear when the average walking distances of the subjects in 6 min were more than 600 m, consisted with Lammers et al. (13) that VO_2max_ is closely related to the 6MWT distance in children with PAH who have a poor exercise capacity (ie, a 6MWT distance below 300 m). Previous studies in adults with cardiovascular problems (14, 15, 27) have shown similar findings, with some deviation in cutoff values. Additionally, when we divided the subjects according to VO_2max_, we identified that the 6MWT did not demonstrate significant importance in the group with higher VO_2max_, or in other words, individuals with a better exercise capacity. This result again supported the earlier finding that submaximal exercise testing is not very effective for this population (13, 28). On the other hand, 6MWT distance and both of the VE/VCO_2_ slope and OUES linearly correlate with no influence of walking distance, probably due to the fact that these two parameters have no certain association with the degree of effort. Therefore, a 6MWT should not be considered the preferred exercise testing modality or a reliable substitute for CPET when assessing the outcome of medical intervention in pediatric patients with relatively mild functional impairment.

In accordance with studies (27–30) it is advised not to conduct the 6MWT in participants younger than 5 years old because of the lack of concentration during 6 min and questionable results in these children. CPET cannot also provide reliable predictive value for participants under 6 years old (31), so there is still a large space for further exploring how to evaluate the exercise capacity of children of this age more objectively.

### Limitations

There were few patients with NYHA III who participated in our study and the population of patients with NYHA IV was not represented in the subjects. As observed in studies including more advanced heart failure, the predictive power of the 6MWT would likely portend additional clinical assessment and prognostic value in these patients. Thus a high sample number of patients with cyanosis or undergone palliative surgery is necessary for this affirmation to be finally consolidated.

This was a cross-sectional analysis and further longitudinal studies are required to assess whether serial CPET or 6MWT represent efficacy endpoints and functional capacity for an individual with CHD.

## Conclusion

Our study confirmed the linear correlation between 6MWT distance and CPET-derived variables in functional impaired pediatric patients with CHD, therefore providing evidence on when a 6MWT should be considered as a convincing complementary test in the pediatric population with CHD. The cutoff values for 6MWT distance may be qualified as one of the intervention targets for exercise rehabilitation.

## Data Availability

The raw data supporting the conclusions of this article will be made available by the authors, without undue reservation.
