# Supplementary material for: Is the six-minute walk test still reliable compared to cardiopulmonary exercise test for exercise capacity in children with congenital heart disease?
Source: Front Pediatr. 2022 Nov 14;10:965739. doi: 10.3389/fped.2022.965739 (PMC9702990; doi:10.3389/fped.2022.965739)
Supplement: Supplementary file 1 [file Datasheet1.doc]

Supplementary materials

**Table S1.** Univariate correlation between 6MWD and the various demographic.

**Table S2.** Confusion matrix of external validation.

**Table S3.** Exercise performance and relationship between peak oxygen uptake and the 6-min walk test in both groups (VO_2max_ ≥80% predicted vs. VO_2max_ <80% predicted)

**Figure S1.** Scatter plots of the relationship between the 6MWT distance and VO_2max_ in children and adolescents with VO_2max_ predicted≥80% with congenital heart disease (A); Scatter plots of the relationship between the 6MWT distance and VO_2max_ in children and adolescents with VO_2max_ predicted <80% with congenital heart disease (B). The red curvy lines in the scatter plots are nonparametric regression.

| Table S1. Univariate correlation between 6MWD and the various demographic. | | | |
| --- | --- | --- | --- |
|  | Total | male | female |
| Age | r=0.280(P<0.001) | r=0.286(P<0.001) | r=0.266(P<0.001) |
| Weight | r=0.393(P<0.001) | r=0.365(P<0.001) | r=0.406(P<0.001) |
| Height | r=0.460(P<0.001) | r=0.424(P<0.001) | r=0.499(P<0.001) |
| BMI | r=0.256(P<0.001) | r=0.227(P<0.001) | r=0.241(P=0.001) |
| BSA | r=0.103(P=0.028) | r=0.115(P=0.066) | r=0.101(P=0.154) |
| 6MWD, six-minute walking distance; BMI, Body Mass Index; BSA, body surface area | | | |

Table S2. Confusion matrix of external validation.

|  |  | Pred% | |
| --- | --- | --- | --- |
|  |  | good | bad |
| 6MWD | good | 43 | 12 |
|  | bad | 13 | 34 |

6MWD, six-minute walking distance

| Table S3. Exercise performance and relationship between peak oxygen uptake and the 6-min walk test in both groups (VO_2max_ ≥80% predicted vs. VO_2max_ <80% predicted) | | | |
| --- | --- | --- | --- |
| Variables | Value | 6MWT |  |
| ***Group1(VO_2max_***≥***80% predicted)*** |  |  |  |
| **Cardiopulmonary exercise testing** |  |  |  |
| VO_2max_ (%pred) | 0.92±0.09 | r=0.04 |  |
| VO_2max_(ml/min/kg) | 39.7±5.2 | r=-0.08 |  |
| **6MWT distance (m)** | 607±50 | - |  |
| ***Group 2 (VO_2max_ <80% predicted)*** |  |  |  |
| **Cardiopulmonary exercise testing** |  |  |  |
| VO_2max_ (%pred) | 0.67±0.10 | r=0.34* |  |
| VO_2max_ (ml/min/kg) | 31.2±5.8 | r=0.17^#^ |  |
| **6MWT distance (m)** | 537±61 |  |  |
| Data are presented as means±SD #:<0.05 *<0.001  VO_2max_, peak oxygen uptake; 6MWT, 6-minute walking test | | | |


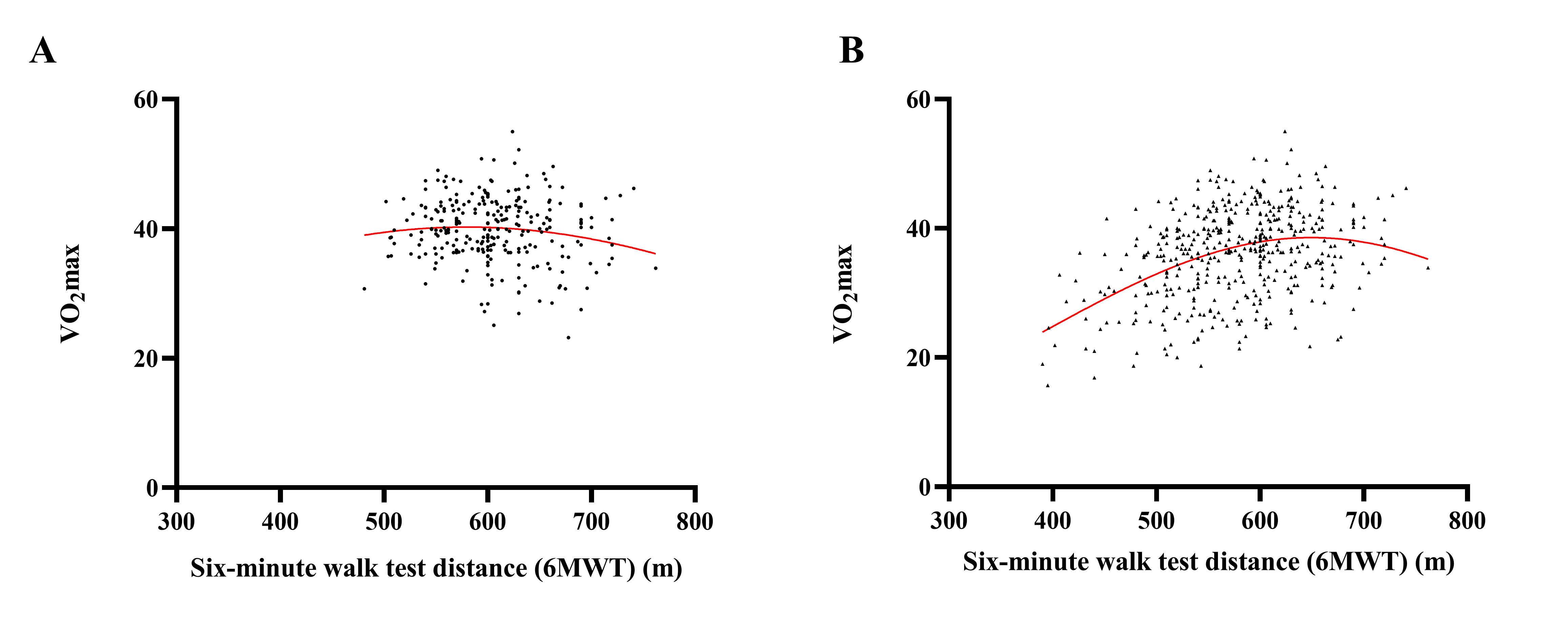


Figure S1. Scatter plots of the relationship between the 6MWT distance and VO_2max_ in children and adolescents with VO_2max_ predicted≥80% with congenital heart disease (A); Scatter plots of the relationship between the 6MWT distance and VO_2max_ in children and adolescents with VO_2max_ predicted <80% with congenital heart disease (B). The red curvy lines in the scatter plots are nonparametric regression.
